# A Preliminary Study of the Carbon Emissions Reduction Effects of Land Use Control

**DOI:** 10.1038/srep36901

**Published:** 2016-11-15

**Authors:** Xiaowei Chuai, Xianjin Huang, Xinxian Qi, Jiasheng Li, Tianhui Zuo, Qinli Lu, Jianbao Li, Changyan Wu, Rongqin Zhao

**Affiliations:** 1 School of Geographic & Oceanographic Sciences, Nanjing University, Nanjing 210023, Jiangsu Province, China; 2 Land Development and Consolidation Technology, Engineering Center of Jiangsu Province, Nanjing 210023, Jiangsu Province, China; 3 Key Laboratory of Development and Protection for the Coastal Zone of the Ministry of Land and Resources, Nanjing 210023, Jiangsu Province, China; 4 Earthquake Administrator of Guangxi Autonomous Region, Nanning, 530022, Guangxi Province, China; 5 North China University of Water Resources and Electric Power, Zhengzhou 450011, Henan Province, China

## Abstract

Land use change not only directly influences carbon storage in terrestrial ecosystems but can also cause energy-related carbon emissions. This study examined spatiotemporal land use change across Jiangsu Province, China; calculated vegetation carbon storage loss caused by land use change and energy-related carbon emissions; analysed the relationship among land use change, carbon emissions and social-economic development; and optimized land use structure to maximize carbon storage. Our study found that 13.61% of the province’s land area underwent a change in type of land use between 1995 and 2010, mainly presented as built-up land expansion and cropland shrinkage, especially in southern Jiangsu. Land use change caused a 353.99 × 10^4^ t loss of vegetation carbon storage loss. Energy-related carbon emissions increased 2.5 times from 1995 to 2013; the energy consumption structure has been improved to some extent while still relying on coal. The selected social-economic driving forces have strong relationships with carbon emissions and land use changes, while there are also other determinants driving land use change, such as land use policy. The optimized land use structure will slow the rate of decline in vegetation carbon storage compared with the period between 1995 and 2010 and will also reduce energy-related carbon emissions by 12%.

Global warming, a problem that is mainly due to greenhouse gas emissions, has already caused significant damage^1^. The warming climate will increase soil respiration^2^ and thus release soil organic carbon SOC into the atmosphere; this will reduce SOC storage, aggravate global warming, and ultimately form a positive feedback cycle^3^. Carbon emissions abatement is an urgent task for most countries and nations, including both developing and developed countries. Land-use changes can affect global carbon budgets significantly by changing the level of carbon storage in the vegetation and soil of terrestrial ecosystems^4^. The main reason for the change in carbon storage is the conversion of vegetation residue to soil, which is the main source for soil organic carbon SOC^5^. The impact of land use change in causing carbon storage loss is second only to the burning of fossil fuels^6^,^7^. It has been reported that global carbon emissions caused by land use change accounted for 20% and 12.5% of total carbon emissions from the 1980s to 1990 and from 2000 to 2009, respectively^8^. Therefore, studies of the impact of land use change on carbon emissions, and of emissions reduction by land-use control, have been carried out by many scholars^9^,^10^,^11^,^12^,^13^. Recent studies show that land-use changes can also affect anthropogenic energy consumption on land surfaces^7^; for example, the expansion of built-up land may attract industrial activity with a high density of energy consumption. Although this result may be not be inescapable, it is a strong possibility^14^.

Due to the significant effect that land-use change can have on carbon emissions, many scholars have explored development that promotes low carbon emissions by way of land use management. Some scholars have studied land use structure optimization based on low carbon emissions, including a study that aimed to maximize terrestrial ecosystem carbon storage^10^,^15^ and another that aimed to minimize energy-related carbon emissions by using land surface as a carrier^16^. Although these studies may offer feasible ways for government to alleviate the pressure to reduce carbon emissions, the implementation process may be difficult and may be not carried out well under the pressure to produce large economic benefits. Land use changes are always driven by economic and social factors^17^,^18^,^19^,^20^. Economic development is also the main driving force behind energy-related carbon emissions^21^,^22^. Land use change and carbon emissions may be linked by their similar driving forces, and to guarantee effective implementation, we believe that land use control and structural optimization need to be carried out from the perspective of controlling these driving factors. Although this may hinder economic benefits to some extent, compared to the potential environmental damages these driving factors could cause, it is highly valuable. Previous studies have not analysed the relationship among land use change, driving forces and energy-related carbon emissions.

It is also important to note that coastal regions always have high population densities, concentrating 60% of the world’s population^9^,^23^. Coastal regions also exhibit obvious land use changes^24^,^25^ and usually have high-intensity energy consumption activities on their land surfaces^16^. These factors make coastal regions an ideal study area typical of land-use change, which may well demonstrate how social-economic development drives both land use change and carbon emissions. Previous studies in Jiangsu Province have usually focused on energy-related carbon emissions^26^,^27^ and terrestrial ecosystem carbon storage changes^5^,^28^,^29^, respectively, while still lacking a comprehensive examination of carbon losses/emissions from both energy consumption and terrestrial ecosystems. Thus, our study intends to fill these gaps. The objectives mainly include the following: 1) examine land use change and its effect on carbon storage change; 2) calculate energy-related carbon emissions; 3) analyse the driving forces of both land use change and energy-related carbon emissions, and link land use change and carbon emissions according to their driving forces; 4) optimize land use structure and major driving factors. Our study will explore a new approach to low carbon development; it makes an essential contribution to current research and will help to guarantee the implementation of low carbon land use.

## Results

### Spatiotemporal land use changes

Total land area increased from 1995 to 2010. Types of land use changes can be summarized as decreasing of cropland, woodland and grassland, and increasing of built-up land and water area. The changes are more obvious between 2005 and 2010 (SI-6, Table S3). Land transformation analysis (Table 1) shows that 13.61% of the land area across Jiangsu Province changed in terms of its use type. A total of 6185.80 km^2^ cropland was occupied by built-up land, accounting for 82.24% of the total area transferred out of cropland. Water is the second type of land use to occupy cropland, but the area transferred to water is much smaller than that transferred to built-up land. The area transferred out of woodland was only 360.20 km^2^, mainly occupied by cropland and built-up land. A total of 51.84% of the province’s grassland has been transferred out, with an area of 893.76 km^2^, including 461.44 km^2^ to water and 130.74 km^2^ to built-up land. Water areas were mainly converted to built-up land and cropland, with 266.21 km^2^ and 159.51 km^2^ converted, respectively. Built-up land was also converted to other land use types, with a total area of 978.68 km^2^ having been converted. Spatially, the transferred area was more obvious in the southern and coastline regions (SI-6, Figure S3).

### Carbon storage loss and carbon emissions

Vegetation carbon densities differed greatly among land use types. Woodland presented the highest value of 2691 t/km^2^, with values of 553, 322, and 132 × 10^4^ t/km^2^ for cropland, grassland and built-up land, respectively. Due to land use changes, total vegetation carbon storage decreased 353.99 × 10^4^ t between 1995 and 2010 (Table 2). Transferring out of cropland, woodland and grassland all led to carbon storage loss, while transferring out of water areas, built-up land and unused land all increased carbon storage. Transferring out of cropland contributed the most to Jiangsu’s total carbon storage loss of 79.76%, with the total amount of lost carbon storage reaching 260.42 × 10^4^ t; cropland-woodland is the only type of transfer to offset carbon storage losses. For woodland, all transfers-out lead to carbon storage loss, with the total amount reaching 85.60 × 10^4^ t, mainly contributed by the transfer to cropland and built-up land. Grassland transferred to cropland and woodland leads carbon storage to increase by 4.68 × 10^4^ t and 0.36 × 10^4^ t, respectively, while transfers to water areas, built-up land and unused land all lead to carbon storage loss. With no vegetation coverage, transfers-out of water areas and unused land all increased carbon storage. For built-up land, the transfer-out to other vegetation-covered land all increased carbon storage (especially for the transition to cropland, which contributed 29.62 × 10^4^ t) while transfers to areas without vegetation coverage and to water areas and unused land decreased carbon storage.

**Figure 1:**
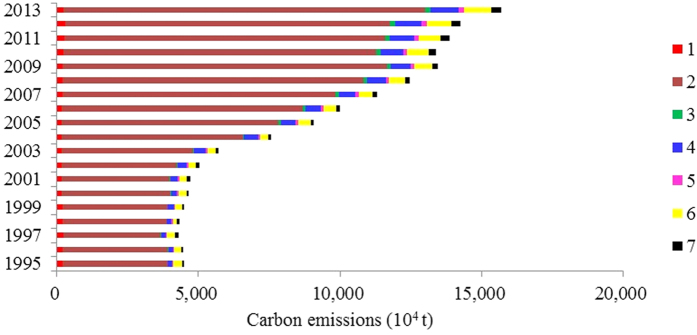
Carbon emissions from energy consumption of different sectors. Numbers 1–7 represent Agriculture, Forestry, Animal Husbandry, Fishery and Water Conservancy; Industry; Construction; Transport, Storage and Post; Wholesale, Retail Trade and Hotel, Restaurants; Residential Consumption; and Others.

Figure 1 shows that total carbon emissions from energy consumption increased from 4475.94 × 10^4^ t in 1995 to 15713.88 × 10^4^ t in 2013, an increase of 2.5 times. The rate of increase is more obvious after 2002. More than 80% of carbon emissions were concentrated in the industrial sector, and the percentage did not change much during 1995–2013, with the lowest value of 80.57% in 2012 and 85.60% in 2007; the amount increased 2.46 times in 2013 compared with 1995. Residential consumption is the second largest carbon emission sector, with percentages fluctuating between 3.96–7.25%. Although absolute emissions from the construction sector are much lower than for other sectors, accounting for only 0.28–1.21% of total carbon emissions in different years, this sector still presented the highest rate of increase (14.11 times). Carbon emissions from the service sectors of “transport, storage and post”, “wholesale, retail trade and hotel, restaurants”, and “others” increased 5.78, 5.7 and 5.34 times from 1995 to 2013, respectively, but still only accounted for small percentages. “Agriculture, forestry, animal husbandry fishery and water conservancy” is the only sector with no obvious increase in carbon emissions, and the percentages accounting for total carbon emissions decreased from 4.69% to 1.46% from 1995 to 2013. Carbon emissions from different energy sources increased between 1995 and 2013, while the energy consumption structure did not change much and continued to rely on coal consumption (SI-7, Figure S4).

### Driving forces of land use changes and carbon emissions

Grassland, water area and unused land all presented irregular trends. Therefore, this study will not present a quantitative analysis of the three land use types and will instead present a separate analysis in the “Discussion” section of this paper. For cropland (*Y*_*1*_), woodland (*Y*_*2*_) and built-up land, there was an obvious linear trend, and thus we conducted a quantitative analysis. We further divided built-up land into residential and industrial land (*Y*_*3*_) and transportation land (*Y*_*4*_) to perform a more detailed analysis. Our correlation analysis (Table 3) shows that 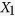
–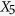 all have significant negative correlations with cropland change (*Y*_*1*_) (P < 0.01), and the correlation coefficients are higher than 0.9, except for X_3_ (−0.892). While for the rural population (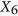), there was a positive correlation with cropland, with a high correlation coefficient of 0.893. Woodland (*Y*_*2*_) also correlated negatively with X_1_–X_5_ and positively with X_6_, while the coefficient values are much lower compared with cropland. Residential and industrial land (*Y*_*3*_) and transportation land (*Y*_*4*_) all correlated significantly and positively with their driving forces at the P < 0.01 level. This means that the social-economic driving forces we selected can effectively explain the increase in built-up land and decrease in ecological land. Carbon emissions (*Z*) have a strong positive relationship with economic and social factors, with high coefficients (between 0.917–0.973) for X_3_, X_2,_ X_4_ and X_1_, and relatively low and negative correlations with X_11_ and X_12_, which means that social and economic development is the main driver of carbon emissions increases; while energy use efficiency is under-promoting and energy structure is under-optimizing, they began to have the effect of restraining carbon emissions increases, although the effect still seems weak.

### Low carbon effect of the optimized land use structure

The optimized land use structure caused woodland and grassland to increase by 1280 km^2^ and 333.33 km^2^, respectively. The decrease in cropland is inevitable; 10,980.5 km^2^ of cropland will be lost compared with 2010 levels, and cropland is the land use type with the largest transferred area. Built-up land will continue to increase by 9582.84 km^2^ to support social and economic development. In total, our optimized land use structure will cause vegetation carbon storage to decrease by 125.55 × 10^4^ t. This is mainly caused by the shrinkage of cropland area, while other land use types can offset this decrease with a land area increase; this is especially true for woodland, which can increase vegetation carbon storage by 344.45 × 10^4^ t, although the increase in woodland area is much lower than for built-up land.

According to Table 4, future land use changes in Jiangsu Province can be represented by decreased cropland and increased built-up land. Here we use these two land use types to establish equations with driving forces. Because the urbanization rate in Jiangsu Province already reached such a high level (above 65%) in 2014 and the population growth rate has slowed in recent years, the linear growth trend may change in the future, the effect on land use change and carbon emissions from population may be weaken. Currently, traditional lifestyles are changing; although the Chinese government announced the two-child policy in 2015, high costs of living, and especially high housing prices, may prevent many couples from having a second child. Extremely high housing prices in many Jiangsu cities, combined with terrible pollution, may also have a negative effect on attracting large populations into cities. Thus, for future predictions, we only select the typical driving forces of GDP (X_1_) and fixed-asset investment (X_2_). According to the established equations (shown below) and optimized land areas of cropland and built-up land in 2030, we can determine that, to guarantee the achievement of the optimized cropland and built-up land areas, GDP and fixed-asset investment should to be controlled at approximately 85% and 90% of the levels based on the linear increasing trend since 1995. According to the calculated GDP and fixed-asset investment in 2030, carbon emissions in 2030 will reach 50111 × 10^4^ t, based on the established equation; this is approximately 3 times the carbon emissions in 2014 but approximately 12% less than what is predicted by GDP and fixed-asset investment according to the linear increasing trend.


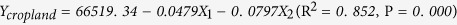



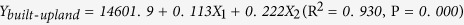



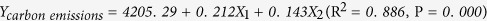


## Discussion

Located on the east coast of China, Jiangsu Province presented obvious land use change between 1995 and 2010. The change was mainly characterized by a continuous expansion of built-up land primarily at the expense of cropland loss, and the trend became increasingly rapid. The economic level of Jiangsu is relatively high compared with other regions^30^, and rapid urbanization and economic development have propelled the expansion of built-up land^18^. Based on the assumption of no carbon gain or loss for all land types between 1995 and 2010, total land use change caused 353.99 × 10^4^ t of carbon storage losses from vegetation; the quantities were much smaller than annual energy-related carbon emissions, although there are still many uncertainties (SI-5). We did not consider the vegetation carbon sink effect during the growing seasons; for example, forests had an obvious carbon sink effect during 2005 and 2010^29^. We also did not consider the effect of SOC because changes in SOC take a longer time to develop than changes in vegetation carbon storage^9^, and the changes in SOC are more complex than those in vegetation. For built-up land expansion, the exclusion of vegetation may prevent plant residues from returning to soil under built-up land, which will reduce soil organic carbon, while the compacted and sealed land cover can also prevent the release of soil carbon into the atmosphere. Because most of the built-up land was converted from ecological land, the SOC value of built-up land may not be too much lower than that of other ecological land-use types^5^, especially in the short term. So, we believe that SOC changes brought about by land use changes require further study. A previous study also showed significant SOC sequestration in the soils of Jiangsu from the 1980s to 2000s, with cropland management playing an important role^28^. Hence, large areas of cropland replaced by built-up land will prevent SOC sequestration due to the sealed land surface. The loss of vegetation carbon storage was mainly due to the loss of cropland, and this will also harm the province’s food security and ability to feed its large population^31^. Although the decreased woodland area is only 3.4% cropland, it contributed the second largest loss of vegetation carbon storage (28% from cropland); this is due to the carbon density of woodland being much higher than all other land use types, as well as the high levels of biomass that this land type sequesters^9^. So, the protection of woodland is the most effective way to increase carbon storage. Regarding spatial differences, land use change in southern Jiangsu was more obvious, and this is consistent with the area’s economic development level^18^. Industries developed faster in these regions and attracted more people to work in the area; thus, more built-up land is needed to feed development, which supports our conclusion that social-economic driving forces are the main power behind land use change in Jiangsu. Social-economic development is also the main driving force behind land use change in other areas^17^,^19^,^20^. Furthermore, based on soil samples covering Jiangsu Province, SOC densities in the surface soil layer of 0–20 cm and the deep layer of 150–200 cm are relatively high compared to most other regions (SI-2, Figure S2). Land use change here will have more obvious potential effects on SOC change, especially for the top soil layer^28^,^32^. Thus, southern Jiangsu faces higher pressure to control built-up land expansion and support ecological land protection.

According to our calculation, energy-related carbon emissions increased 2.5 times during 1995–2010, while GDP increased 4.88 times based on 1995 constant prices. This indicates that carbon emission efficiency has been improved and began to have a negative effect on carbon emissions increases. Efficiency needs to be reexamined using more effective indicators of physical emissions efficiency, such as emissions per unit of steel production. Such indicators are more reactive and can be used to measure the efficiency of different sectors^33^. The carbon emission coefficients used in our study are taken from the IPCC, a recent paper published in “Nature” found that the emission coefficient of coal in China is approximately 40% percent lower than the IPCC value^34^. If this is reliable, then actual energy-related carbon emissions may be much lower than our calculated values because coal is always used as the main energy source in Jiangsu; although its contribution to total carbon emissions dropped from 65.51% to approximately 40% in recent years. This drop may be an improvement to Jiangsu’s energy consumption structure and a positive signal of future reductions in carbon emissions; just as carbon emissions in the US peaked in 2007, the decline after 2007 has been widely attributed to a shift from the use of coal to natural gas in US electricity production^21^. Unlike in Jiangsu and the rest of China, around the world, oil consumption has already exceeded coal consumption^35^, so, improvements to the energy consumption structure in Jiangsu and China are needed, especially in terms of the development of clean and renewable energy, to reduce both carbon emissions and other contaminants. Analysis of driving forces also shows that the increase in carbon emissions in Jiangsu was also mainly linked to economic development, as in the rest of China^36^,^37^ and in other countries^22^,^38^. This is because, to feed its large population and social-economic development, China still relies on the production of secondary industry, and these production processes rely on energy consumption and thus cause major carbon emissions^14^. According to GDP data from the Jiangsu statistical book, secondary industry has always been the anchor for Jiangsu’s economy, accounting for approximately 50% of total GDP. Secondary industry has always contributed more than 80% of total energy-related carbon emissions from 1995–2013, which means that Jiangsu’s economic structure still relies on secondary industry, economic structural improvement is necessary for low carbon development. According to our analysis of the driving forces behind land use change, in addition to social-economic development, land use change may also be affected by other exterior determinants, for example, the non-obvious decreasing trend in woodlands may benefit from a forest protection policy in Jiangsu, such as afforestation and the establishment of natural conservation areas. Water areas present an increasing trend, which is most obvious between 2000 and 2005; this is due to the development of aquaculture, as many land areas have been excavated as fish ponds^39^. Although grassland presents a decreasing trend, while, its area has increased dramatically in recent years, mainly due to the Jiangsu government’s coastal land use plan to plant 333 km^2^ of grass area artificially in coastal Jiangsu^16^. And due to the natural sediment effect, the tideland reclamation policy also had an important effect on accelerating total unused land (shallow area)^9^,^39^. Thus, land use policy, regional development strategies and physical effects can also have obvious effects on land-use changes.

China has promised to reach its carbon emission peak in approximately 2030^14^. As a developed region in China, Jiangsu Province makes a major contribution to the country’s GDP, and it has risen to become the second-highest contributor (only slightly below Guangdong Province in 2014 according to the China Statistical Yearbook). So, under the pressure of economic development, land use changes and carbon emissions control in Jiangsu faces a major challenge. Many measures have been proposed to reduce energy-related carbon emissions, such as building a carbon trading system^33^, improvements to energy structure^21^, a carbon tax^40^, carbon capture^41^, carbon dioxide storage^42^, etc. While our study has linked land use change and carbon emissions by social-economic driving forces, explored the effect of land use control both on carbon storage directly and on energy-related carbon emissions indirectly, it is the first attempt to address these issues that may have some negative effects on economic development. Chinese government has often made environmental protection plans, such as land use plans, that have been ineffective, such as the land use plan of 2005 in Jiangsu Province that aimed to limit built-up land expansion to 877.34 km^2^. However, the area of built up land actually increased to 4.89 times what was originally planned. Plans that are out of touch with the economic baseline seem difficult to implement and complete because advanced green technology and low carbon strategies are difficult to improve in a short time in China. Under our optimized land use structure, vegetation carbon storage will lose 125.55 × 10^4^ t between 2010–2030, only 35% of what was lost during the period 1995–2010; this indicates an effective land use structure that will maximize carbon storage. According to our simulation, to guarantee the implementation of the optimized land use structure, Jiangsu may lose approximately 15% of GDP and 10% of fixed-asset investment compared with the trend of free development, but meanwhile, energy-related carbon emissions will be reduced by 12%. And in addition to reducing carbon emissions, the protection of ecological land can also offer many other ecological benefits, such as the regulation of the regional climate, the provision of food and water, and the protection of biodiversity^43^,^44^. Therefore, this land use structure optimization is urgently needed; although it will harm rapid economic development to some extent, we believe that environmental protection will bring more potential benefits than economic losses.

The 2030 prediction also has some uncertainties. First, during this period, technology, the economic structure and the energy consumption structure may be improved, which will greatly affect carbon emissions quantities. Second, most local development has not been planned for 2030, there are few policy references for us to consult, many land use changes were predicted based on assumptions or historical trends, and thus the land use change in our study has large uncertainties compared with our previous studies of the year 2020^9^,^10^,^16^. The predictions of GDP and fixed-asset investment were based on the established equations and the optimized land use area in 2030; equation establishment will greatly affect our prediction results. Our study shows that GDP and fixed asset investment in 2030 will increase 5.38 and 4.1 times compared with 2010, while the historical increase rates are 4.88 and 9 times between 1995 and 2010. Fixed-asset investment seems to introduce larger uncertainties, or in other words, comes at the cost of contributing to land use structure optimization and carbon emissions reduction. Matched by the optimal land use structure-based economic level, energy-related carbon emissions in 2030 increased 2.7 times; this increase rate seems reasonable because from 1995 to 2010, total energy-related carbon emissions increased 2 times in 15 years. Overall, although there are many uncertainties, there is hope that our predictions may be achieved, as they will not harm the economy to a great extent.

Our study examined land use change, vegetation carbon storage loss and energy related carbon emissions in China in a typical region of Jiangsu Province. It made the first step toward linking land use change and carbon emissions by driving forces. The analysis of this relationship may be simple, but it also provides a totally new insight into low carbon development strategies and policies. We believe that further studies are needed to provide more deep and complex insights into the relationship among land use change, energy-related carbon emissions and social-economic development and to help make more accurate predictions.

## Method

### Study area

**Figure 2:**
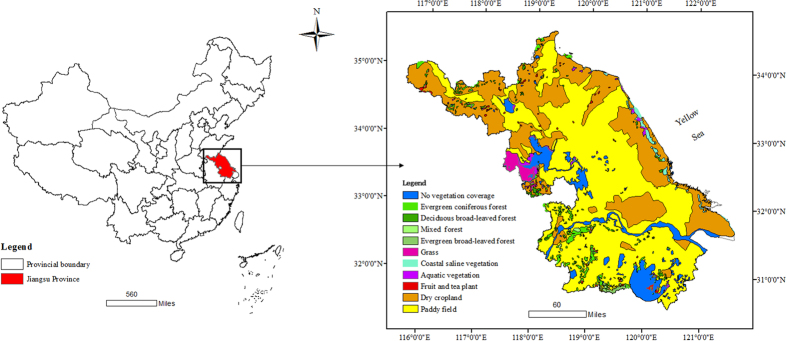
Location of Jiangsu Province and main vegetation coverage. Map created using ArcGIS [9.3], (http://www.esri.com/software/arcgis).

Jiangsu Province is located in eastern China, facing the Yellow Sea (Fig. 2). Annual temperature is approximately 13.6–16.1 °C, and annual rainfall is approximately 1,000 mm; 85% of the terrain is plains. It has diverse natural vegetation types but only accounts for 6% of total regional area. The large area of crop vegetation accounts for more than 80% of the whole regional area. The economic level in Jiangsu is high compared to the rest of China, and the Gross Domestic Product (GDP) per capita is higher than all other 31 provinces in China^30^.

### Data sources

Data sources included land-use data, soil sample data, forest resource survey data, crop yield data, social and economic data, energy consumption data, carbon emission coefficients, and some empirical data. Land images with a spatial resolution of 30 m are produced from the original data sources of Landsat (TM/ETM+/CBERS) with time series of 1995, 2000, 2005, and 2010. The land-use classifications include 6 first-level classifications and 25 second-level classifications (SI-1, Table S1, Figure S1). The statistical data of land use structure from 1995–2010 are provided by the Land and Resources Department of Jiangsu Province. The vegetation-type map was compiled using data from the 2000s and was able to effectively describe the most recent vegetation distribution in the study area. The more than 20000 soil samples uniformly distributed across Jiangsu Province were obtained from a multi-purpose regional geochemical survey in Jiangsu Province after the year 2000^5^. Forest data were acquired from the Fifth Forest Resource Inventory in Jiangsu province. Crop yield data and other eco-social data were obtained from statistical yearbooks of Jiangsu Province. Other empirical data were obtained from related research studies. Energy consumption data were obtained from the “China Energy Statistical Yearbook” and “Jiangsu Statistical Yearbook”. The per unit calorific values are mainly from the China Energy Statistical Yearbook and are partly from the Intergovernmental Panel on Climate Change (IPCC)^45^. Carbon emission coefficients were from the IPCC (2006). Social and economic data were from the “Jiangsu Statistical Yearbook”.

### Methods

Vegetation-covered lands include woodland, cropland, and grassland; their vegetation carbon densities are calculated by mean biomass density and carbon content of vegetation bodies; detailed calculations are shown in SI-2. Then, according to the calculated vegetation carbon densities and land transfers, carbon storage change can be calculated (SI-2). Energy-related carbon emissions were calculated by energy consumption amount and carbon emission coefficients (SI-3). The Linear Programming Model was used to adjust the land use structure. Application of this model included establishing the target function and constraint conditions. The target was formulated as a maximum or minimum, and the constraint conditions included several control variables. Details on the model used in this study are shown in SI-4. For the analysis of social-economic driving forces of land use change and carbon emissions, correlation and regression methods were used. Other driving forces of land use change are discussed in the “Discussion” section.

## Additional Information

**How to cite this article**: Chuai, X. *et al.* A Preliminary Study of the Carbon Emissions Reduction Effects of Land Use Control. *Sci. Rep.* **6**, 36901; doi: 10.1038/srep36901 (2016).

**Publisher’s note:** Springer Nature remains neutral with regard to jurisdictional claims in published maps and institutional affiliations.

## Supplementary Material

Supplementary Information

## Figures and Tables

**Table 1:**
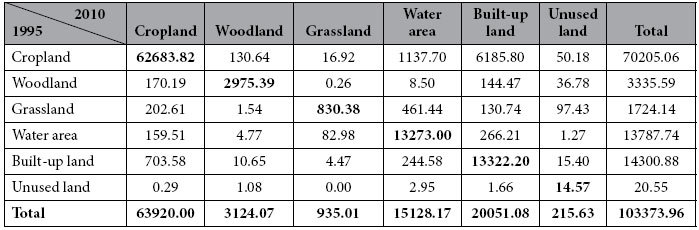
Land transformation matrix of Jiangsu Province between 1995 and 2010 (km^2^).

**Table 2:**
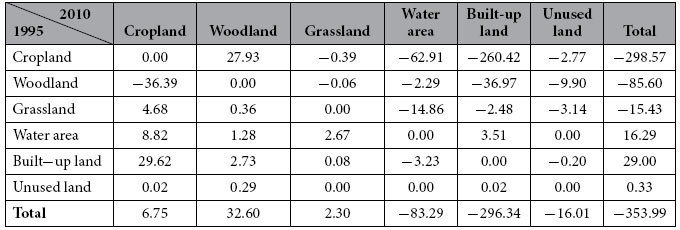
Vegetation carbon storage change based on land transformation matrix between 1995 and 2010 (×10^4^ t).

**Table 3:** Results of correlation analysis between typical land use change and driving forces.

Driving forces		Cropland	Woodland	Residential and industrial land	Transportation land	Carbon emissions
		Y_1_	Y_2_	Y_3_	Y_4_	Z
Gross domestic product (GDP)	X_1_	−0.919^**^	−0.715**	0.984**	0.957**	0.973**
Fixed-asset investment	X_2_	−0.917^**^	−0.739**	0.986**	0.974**	0.943**
Urbanization rate	X_3_	−0.892^**^	−0.607**	0.917**	0.873**	0.917**
Total population	X_4_	−0.915^**^	−0.578*	0.954**	0.884**	0.961**
Urban population	X_5_	−0.903^**^	−0.584*	—	—	—
Rural population	X_6_	0.893^**^	0.575*	—	—	—
Urban per capita housing area,	X_7_	—	—	0.967**	—	—
Rural per capita housing area,	X_8_		—	0.927**	—	—
Annual passenger capacity	X_9_	—	—	—	0.917**	—
Annual cargo capacity	X_10_	—	—	—	0.934**	—
Carbon emissions per capital GDP	X_11_	—	—	—	—	−0.632**
Percentage of coal in energy consumption	X_12_	—	—	—	—	−0.642**

Significant at ^∗^*P* = 0.05 and **P = 0.01 levels.

**Table 4:** Comparison of the optimized land use structure and land use in 2010.

Land use type	Land use area (km^2^)			Vegetation carbon storage (10^4^t)		
	2010	2030	Changes	2010	2030	Changes
Cropland	63935.24	52954.74	−10980.50	3535.62	2928.40	−607.22
Woodland	3126.75	4406.75	1280.00	841.41	1185.86	344.45
Grassland	935.28	1268.61	333.33	30.12	40.85	10.73
Water area	15825.84	15825.84	0.00	0.00	0.00	0.00
Built-up land	20060.67	29643.51	9582.84	264.80	391.29	126.49
Unused land	215.67	0.00	−215.67	0.00	0.00	0.00
Total	104099.45	104099.45	0.00	4671.94	4546.40	−125.55
