# Supplementary material for: A Preliminary Study of the Carbon Emissions Reduction Effects of Land Use Control
Source: Sci Rep. 2016 Nov 15;6:36901. doi: 10.1038/srep36901 (PMC5109288; doi:10.1038/srep36901)
Supplement: Supplementary Information [file srep36901-s1.doc]

**Supporting Information**

Manuscript Title

**A Preliminary Study of the Carbon Emissions Reduction Effects of Land Use Control**

Xiaowei Chuai^1^, Xianjin Huang^1, 2, 3^, Xinxian, Qi^1^, Jiasheng Li^1^, Tianhui Zuo^4^, Qinli Lu^1^, Jianbao Li^1^, Changyan Wu^1^, Rongqin Zhao^5^

^1^School of Geographic & Oceanographic Sciences, Nanjing University, Nanjing 210023, Jiangsu Province, China. ^2^Land Development and Consolidation Technology, Engineering Center of Jiangsu Province, Nanjing 210023, Jiangsu Province, China. ^3^Key Laboratory of Development and Protection for the Coastal Zone of the Ministry of Land and Resources, Nanjing 210023, Jiangsu Province, China. ^4^Earthquake Administrator of Guangxi Autonomous Region, Nanning, 530022, Guangxi Province, China. ^5^North China University of Water Resources and Electric Power, Zhengzhou 450011, Henan Province, China

Correspondence and requests for materials should be addressed to X.W.C. (chuaixiaowei@163.com) or X.J.H. (hxj369@nju.edu.cn)

# SI-1. Land use data

Land images with a spatial resolution of 30 m are produced from the original data sources of Landsat (TM/ETM+/CBERS), with time series of 1995, 2000, 2005, and 2010. The land-use classifications include 6 first-level classifications and 25 second-level classifications (Table S1). With these basic data (Figure S1), spatiotemporal changes of different land-use types can be analysed.

Table S1 Land-use classifications system of the Chinese Academy of Science

| First-level classifications | Second-level classifications |
| --- | --- |
| Cropland | Paddy field |
|  | Dry cropland |
| Woodland | Forest land |
|  | Shrub land |
|  | Sparse woodland |
|  | Other woodland |
| Grassland | Highly covered grassland |
|  | Moderately covered grassland |
|  | Lowly covered grassland |
| Water area | River and canal |
|  | Lake |
|  | Reservoir, pond |
|  | Glaciers and permanent snow |
|  | Shallow |
|  | Beach land |
| Built-up land | Urban land |
|  | Rural residential land |
|  | Industry and traffic used land |
| Unused land | Desert |
|  | Gobi |
|  | Saline and alkaline land |
|  | Swampland |
|  | Bare land |
|  | Rock and gravel land |
|  | Other unused land |


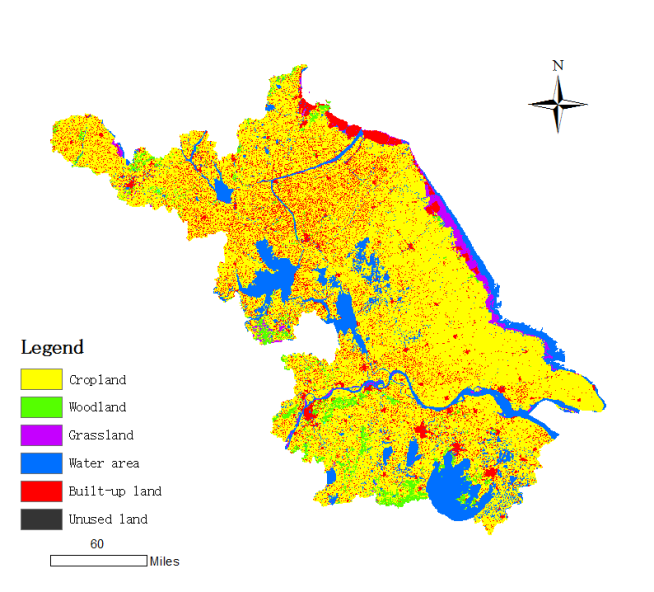

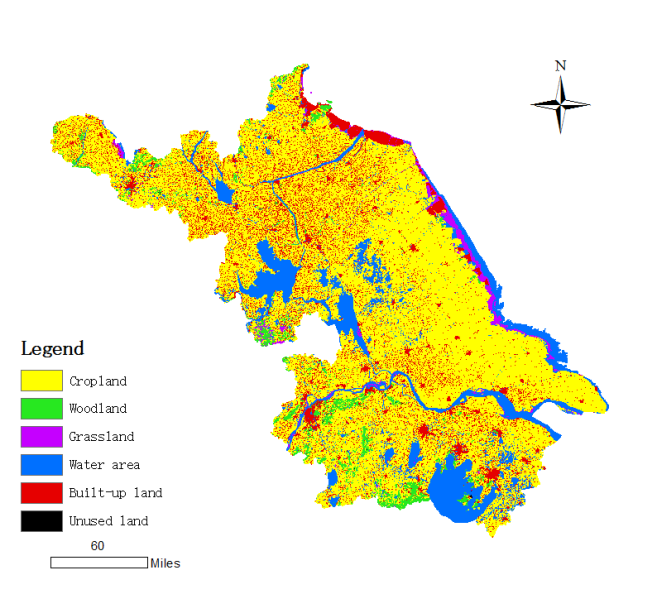


1995 2000


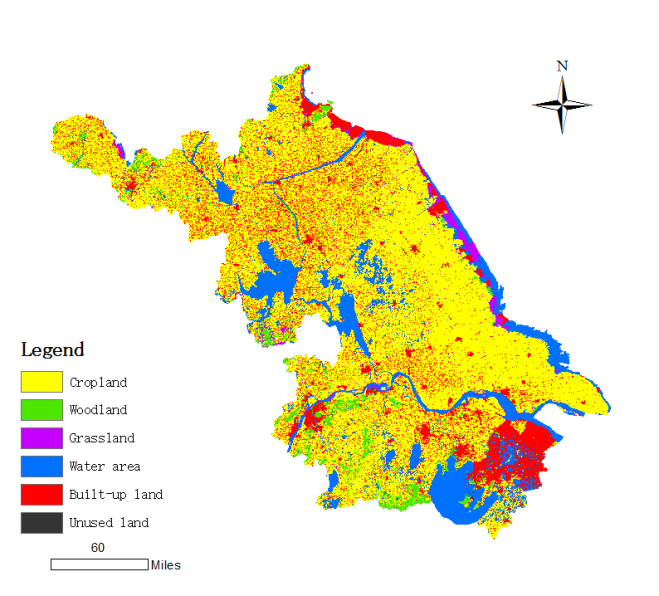

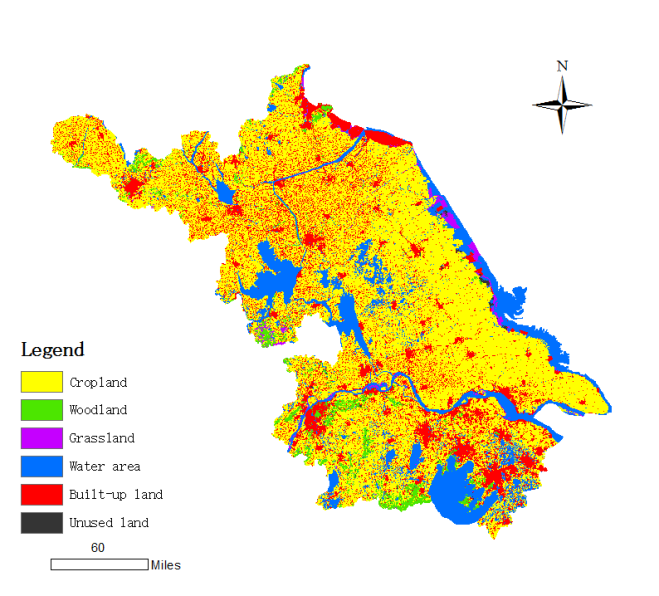


2005 2010

Figure S1 The 30× 30 km grid land-use map of China in 1995, 2000, 2005 and 2010, with the first-level classifications of cropland, woodland, grassland, water area, built-up land and unused land. Map created using ArcGIS [9.3], (http://www.esri.com/software/arcgis)

# SI-2. Carbon densities of vegetation and soil

***Vegetation carbon densities for different land uses***

1) Woodland

We use biomass density and its carbon content to calculate the vegetation carbon density of woodland. Carbon content values of trees are usually defined as 0.45-0.5(Chuai et al., 2014) 5internationally and as 0.5 in China; here we define this value as 0.5. The calculation process is shown below:

$W=\frac{\sum_{ij} W_{ij}\times S_{ij}}{\sum_{ij} S_{ij}}$ $W_{ij}\text{=B}_{ij}\times0.5$ $B_{ij}=a+bV_{ij}$ (S1)

$W$represents the average vegetation carbon density of woodland, $W_{ij}$, $S_{ij}$and $B_{ij}$represent vegetation carbon density; woodland area and vegetation biomass density for tree-type$i$and tree-age$j$, respectively. $V_{ij}$is tree volume density, which can be obtained from the Fifth Forest Resource Inventory in Jiangsu; $a$and $b$are constant values obtained from the study result of Xu et al. (2007), who established linear regression equations between forest biomass and its volume based on 2304 forest sample plots in China.

2) Cropland

Main crops planted in coastal Jiangsu include rice, wheat, corn, beans, potato, cotton, peanuts, rapeseed, sugar and vegetables. Here we calculate vegetation density according to their yields (Piao et al., 2009) (Formula S2):

$C=\frac{\sum_{i} C_{i}\times S_{i}}{\sum_{i} S_{i}}$ $C_{i}=\frac{P_{i}\times(1-W_{i})\times Y_{i}}{H_{i}\times S_{i}}$ (S2)

$C$is the average vegetation carbon density of crop land. $C_{i}$represents the vegetation carbon density of crop type $i$; $S_{i}$ is the area of crop type $i$;$P_{i}$, and$H_{i}$represent crop yield , water content, the ability to absorb carbon and the [economic coefficient](http://dict.baidu.com/s?wd=economic coefficient ) of crop type $i$, respectively. $S_{i}$ and$P_{i}$were obtained from the Jiangsu Statistical Yearbook, and the values of $W_{i}$, $Y_{i}$and$H_{i}$were obtained from the related study of Zhao (2011).

3) Other land

For grassland, we use the same method of calculating biomass as used for woodland to calculate its vegetation carbon density, as grassland is mainly located along the coastline; the biomass data for different vegetation types are taken from the research of Zong et al.(1992), who studied the biomass of salt vegetation and sand vegetation along the coast of Jiangsu Province. According to the vegetation type map, we can calculate the area for each vegetation type, and then the average vegetation carbon density of grassland can be calculated. For built-up land, because trees and grass are scattered among its covered area, its vegetation carbon density was determined according to the vegetation coverage rate and the mean vegetation carbon density values of woodland and grassland. For water area and unused land, we defined their vegetation carbon density as 0 because they have nearly zero vegetation coverage.

***Vegetation carbon storage loss from land use change***

According to calculated vegetation carbon densities and areas of land transfer, carbon storage change can be calculated as follows:

(S3)

Where $C_{ij}$represents vegetation carbon storage change from land use transfer of type $i$ to $j$, $V_{i}$and $V_{j}$ are vegetation carbon densities of land use types $i$ and $j$, respectively, and is transferred land area of land use type $i$ to $j$.

***SOC densities across Jiangsu Province***

There were more than 20,000 soil samples uniformly distributed across Jiangsu Province, obtained from a multi-purpose regional geochemical survey in Jiangsu Province after year 2000. Each soil sample has a record of latitude and longitude, soil organic carbon content (%), soil type, and bulk density. Detailed SOC testing and disposal processes can be obtained from the study of Liao et al. (2009), including data on both the surface soil layer (0-20cm) and the deep soil layer (150-200cm). To compute the spatial variability of SOC densities, we first produced a soil sample distribution map according to the latitude and longitude of each soil sample, and then Kriging methods (based on a spherical model) were employed, and the sample point data were converted to polygon data covering the entire study area (Figure S2).


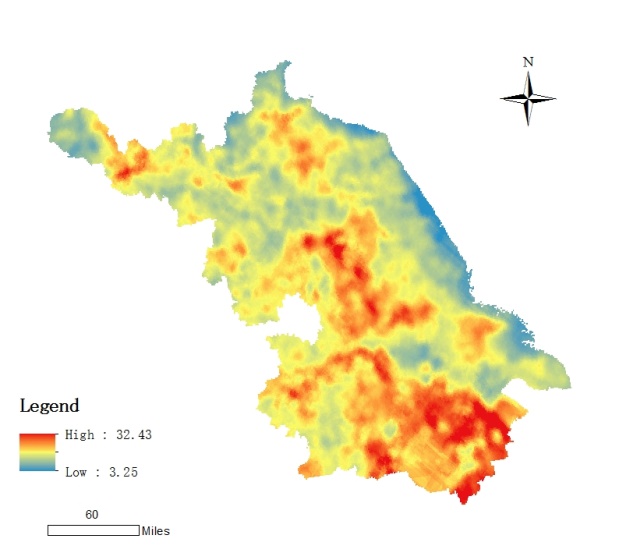

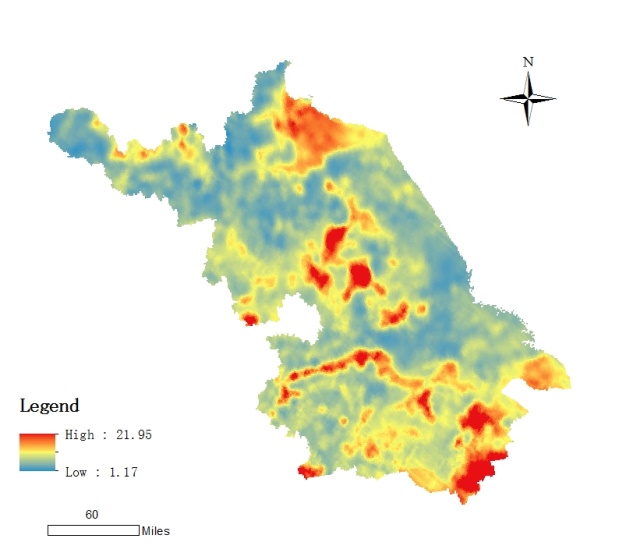


(a) 0-20 cm soil layer (b) 150-200 cm soil layer

Figure S2 Spatial distribution of SOC densities of different soil layers (kg/m^3^) across Jiangsu Province. Map created using ArcGIS [9.3], (http://www.esri.com/software/arcgis)

# SI-3. Energy-related carbon emissions

Energy-related carbon emissions were calculated using Formula S4:

$C_{i}=Q_{i}\times K_{j}\times(V_{CO_{2}-i}+V_{CH_{4}-i})$ (S4)

Where $C_{i}$ is the quantity of carbon emitted by energy type $i$, $Q_{i}$ is the quantity of energy consumption of type $i$, $K_{i}$ is the per unit calorific value of energy type $i$, and $V_{CO_{2}-i}$and $V_{CH_{4}-i}$ are the carbon emission coefficients of CO_2_ and CH_4_ from energy $i$, respectively (Table S2).

The combustion energy consumption data were obtained from the “China Energy Statistical Yearbook”. Energy consumption data mainly include coal, coke, crude oil, gasoline, kerosene, diesel oil, fuel oil, natural gas and electricity, etc. In the calculation of carbon emissions, parameters of per unit calorific value and the carbon emission coefficients of CH_4_ and CO_2_ are needed for each energy type. Per unit calorific values are mainly from the China Energy Statistical Yearbook, and for some energy types (coal products, briquettes, other coking products and coke oven gas) that are missing from the “China Energy Statistical Yearbook”, values are quoted from the Intergovernmental Panel on Climate Change (IPCC) (2006). Carbon emission coefficients of CO_2_ and CH_4_ are quoted from the IPCC (2006). The details are shown in Table S2.

Table S2 Main energy type and carbon emission coefficients

| Energy type | Per unit calorific value | | Carbon emission coefficients of CO_2_ | Carbon emission coefficients of CH_4_ | Calculated total carbon emission coefficients | |
| --- | --- | --- | --- | --- | --- | --- |
|  | Value | Unit | （kg C/GJ） | (×10^-3^.kg C/GJ) | Value | Unit |
| Raw coal | 20908 | KJ/kg | 25.80 | 0.75 | 0.5394 | kg/kg |
| Cleaned coal | 26344 | KJ/kg | 26.21 | 0.75 | 0.6905 | kg/kg |
| Washed coal | 9409 | KJ/kg | 26.95 | 0.75 | 0.2536 | kg/kg |
| Coal products | 15910 | KJ/kg | 26.60 | 0.75 | 0.4232 | kg/kg |
| Briquettes | 9409 | KJ/kg | 26.60 | 0.75 | 0.2503 | kg/kg |
| Coal water slurry | 9409 | KJ/kg | 26.95 | 0.75 | 0.2536 | kg/kg |
| Pulverized coal | 9409 | KJ/kg | 26.95 | 0.75 | 0.2536 | kg/kg |
| Coke | 28435 | KJ/kg | 29.20 | 0.75 | 0.8303 | kg/kg |
| Other coking products | 34332 | KJ/kg | 26.60 | 2.25 | 0.9132 | kg/kg |
| Coke oven gas | 17354 | KJ/m^3^ | 12.10 | 0.75 | 0.2100 | kg/m^3^ |
| Blast furnace gas | 2985 | KJ/m^3^ | 70.80 | 0.75 | 0.2114 | kg/m^3^ |
| Other gas | 16970 | KJ/m^3^ | 60.20 | 0.75 | 1.0216 | kg/m^3^ |
| Natural gas | 38931 | KJ/m^3^ | 15.30 | 0.75 | 0.5956 | kg/m^3^ |
| Crude oil | 41816 | KJ/kg | 20.00 | 2.25 | 0.8363 | kg/kg |
| Gasoline | 43070 | KJ/kg | 18.90 | 2.25 | 0.8140 | kg/kg |
| Kerosene | 43070 | KJ/kg | 19.60 | 2.25 | 0.8442 | kg/kg |
| Diesel oil | 42652 | KJ/kg | 20.20 | 2.25 | 0.8616 | kg/kg |
| Fuel oil | 41816 | KJ/kg | 21.10 | 2.25 | 0.8823 | kg/kg |
| Liquefied petroleum gas | 50179 | KJ/kg | 17.20 | 0.75 | 0.8631 | kg/kg |
| Refinery gas | 46055 | KJ/kg | 15.70 | 0.75 | 0.7231 | kg/kg |
| Coal tar | 33453 | KJ/kg | 20.00 | 2.25 | 0.6691 | kg/kg |
| Other petroleum products | 37681 | KJ/kg | 20.00 | 2.25 | 0.7536 | kg/kg |
| Electricity | 1 | KJ/kJ | 26.95 | 0.75 | 0.0000 | kg/kg |
| Heat | 3596 | KJ/KWh | 26.95 | 0.75 | 0.0969 | kg/kwh |

# SI-4. Land use structure optimization

LINGO software was used to complete the optimization, details are shown below:

$T=\sum_{i=1}^{n} A_{i}V_{i}=\max$ $i=1,2,3,...,n$. (S5)

Where $T$is regional total vegetation carbon storage; $A_{i}$is the area of land use type$i$, and $V_{i}$is the vegetation carbon density of land use type$i$.

The study established constraint conditions for 6 variables as follows: cropland$X_{1}$, woodland$X_{2}$, grassland$X_{3}$, water area$X_{4}$, built-up land$X_{5}$, and unused land$X_{6}$. We used 2010 as the initial year and 2030 as the target prediction year.

Total land area in Jiangsu Province is increasing due to the sediment effect. The increasing area will be determined by complex factors of both human disturbance and physical effects and is therefore hard to predict. Here we will not consider total land area change in 2030, and we used the 104099.45 km^2^ of 2010 as the constant value. So, the first constraint condition can be established as follows:

$\sum_{i=1}^{6} X_{i}=104099.45\begin{matrix} & \end{matrix}X_{i}\geq0$ (S6)

Cropland continually decreased between 1995 and 2010, and the decreasing trend clearly accelerated, especially for the period of 2005-2010, with the area decreasing by 3586.1 km^2^. With urbanization and social-economic development, this decreasing trend is difficult to change. If we predict cropland area based on the decreasing speed between 2005 and 2010, 49590.84 km^2^ of cropland will be left in 2030, while the speed may be slowed if it is well controlled. So, we gave it a low value. We established an equation between cropland area and the time series of 1995-2010, and according to this equation there will be 52954.74 km^2^ of cropland in 2030. Because the decreasing trend is accelerating to meet the requirements of urbanization and social-economic development, the real area occupied by cropland may be higher than this; so, we set 52954.74 km^2^ as the high value.

$49590.84\leq X_{1}\leq52954.74$ (S7)

The latest land use plan of Jiangsu Province aimed to increase woodland area by 854.55 km^2^ in 2010 compared with 2005, while the actual situation is that the woodland area in 2010 decreased by 355.43 km^2^ compared to 2005; woodland protection thus faces a major challenge. In the future, woodland protection must be strengthened, and at the very least, the decreasing trend should be stopped. We used a woodland area of 3126.75 km^2^ in 2010 as our low value target. The Jiangsu land use plan aimed to increase woodland area to 1280 km^2^ in 2020 compared with 2010; we assume this target may require more time and may be finished in 2030; we therefore set the high value of 4406.75 km^2^ in 2030.

$3126.75\leq X_{1}\leq4406.75$ (S8)

The historical decrease in grassland was mainly due to conversions to water areas for aquaculture. However, the demand for aquaculture may decline in the future as the industrial structure changes, and the decreasing grassland trend will hopefully be stopped in the future. Here we set the grassland area in 2010 as our low value. According to the remote sensing data in our study, grassland here includes herbaceous plants along the coastline, mainly distributed in the coastal city of Yancheng. According to Yancheng City Coastal Agricultural Development Planning, 333.33 km^2^ of grass will be planted in coastal Yancheng by 2020; however, this target seems ambitious when considering the economy and development of tourism. We assume the 333.33 km^2^ target will be completed in 2030 and use it as our high value:

$935.28\leq X_{3}\leq1268.61$ (S9)

Jiangsu Province is rich in precipitation, and natural shrinkage of water areas is difficult. Compared with the period of 2000-2005, the increasing growth of water areas slowed down between 2005 and 2010. Since the demand for aquaculture will decline in the future as discussed above, water area expansion will be prohibited to some extent, and the rate of increase may be lower than between 2005-2010. However, water areas in our method of land classification also include water conservation facilities and land, and we believe that more water conservation will be implemented until 2030 for modern agricultural development. So, total water area may continue to increase, and the water area in 2030 should, at least, be no less than in 2010, 15825.84 km^2^, and should it be lower than the 17571.28 km^2^ calculated using the rate of increase during the period of 2005-2010.

$15825.84\leq X_{4}\leq17571.28$ (S10)

The latest land use planning by the government aimed to limit the increase in built-up land to 877.34 km^2^ and 1423 km^2^ between 2005-2010 and 2010-2010, respectively. However, our study shows that actual built-up land reached 4286.22 km^2^ between 2005 and 2010, which is 4.89 times what was planned. The control of built-up land expansion faces high pressure because of the demands of social-economic development. Here we assume strict measures will be taken to control the rapidly increasing rate, and we predict built-up land area according to the rate of increase between 2010 and 2020 (as the land use plan predicts), which is 22906.67 km^2^; we set this as the low value. We assume twice the rate of increase between 2010 and 2020 to predict the built-up land area in 2030, 25752.67 km^2^, and we set this as the high value.

$22906.67\leq X_{5}\leq25752.67$ (S11)

Unused land accounts for little area; we assume no unused land will be left in 2030.

# SI-5. Limitations and assumptions

Our study contains uncertainties. First, the carbon densities of vegetation are not constant values; In our study, due to data limitations, we did not consider the carbon sink/source effect of vegetation; the only changes in carbon storage that we considered were changes resulting from the conversion of one land-use type to another, and our study only considers carbon emissions from land-use changes; the effects of land-cover changes, including changes in land management, are not considered. Second, in the calculation of energy-related carbon emissions, as with most other studies in China, we mainly used emissions coefficients from IPCC (SI-3, Table S2); this calculation may not accurately reflect the actual situation of Jiangsu Province. Third, during the prediction process for 2030, other factors that can greatly change trends in carbon emissions, such as technology, improvements to industrial structure, carbon taxes and trading strategies, energy consumption structure, etc. were not considered. Fourth, the satellite land use images data may not completely match the statistical land use data.

# SI-6. Land use area and changes

Table S3 shows that total land area in Jiangsu increased 696.07 km^2^ according to the 30m grid land use image, which is mainly from the unused land. The largest land use type is cropland, accounting for about 67.91%-61.42% of the whole study area between 1995 and 2010. Cropland kept decreasing continuously and then accelerated, with the area decreasing 87.52km^2^/year, 451.57 km^2^/year and 717.22 km^2^/year, during the periods of 1995-2000, 2000-2005, and 2005-2010, respectively. Jiangsu is rich in water resources, with water areas on land surfaces accounting for 13.34% in 1995 and increasing to 15.2% in 2010; the total area increased 2030.95 km^2^, most of which occurred between 2000-2005. Built-up land changed the most, increasing 5758.64 km^2^, and the rate of increase is speeding up, especially for the period 2005-2010, with an annual rate of increase of 857.24 km^2^/year. Woodland and grassland areas in Jiangsu presented relatively low area, but both decreased between 1995 and 2010, with areas of 3344.20 km^2^ and 1724.93 km^2^ in 1995 km^2^ decreasing to 3126.75 km^2^ and 935.28 km^2^, respectively.

Table S3 Area changes for different land use types of Jiangsu Province in different years (km^2^)

| Land use type | 1995 | 2000 | 2005 | 2010 | 1995-2000 | 2000-2005 | 2005-2010 | 1995-2010 |
| --- | --- | --- | --- | --- | --- | --- | --- | --- |
| Cropland | 70216.78 | 69779.17 | 67521.34 | 63935.24 | -437.61 | -2257.83 | -3586.1 | -6281.54 |
| Woodland | 3344.20 | 3382.68 | 3482.18 | 3126.75 | 38.48 | 99.50 | -355.43 | -217.45 |
| Grassland | 1724.93 | 1488.77 | 1287.18 | 935.28 | -236.16 | -201.59 | -351.90 | -789.65 |
| Water area | 13794.89 | 14066.22 | 15389.48 | 15825.84 | 271.33 | 1323.26 | 436.36 | 2030.95 |
| Built-up land | 14302.03 | 14668.51 | 15774.45 | 20060.67 | 366.48 | 1105.94 | 4286.22 | 5758.64 |
| Unused land | 20.55 | 19.70 | 18.29 | 215.67 | -0.85 | -1.41 | 197.38 | 195.12 |
| **Total** | 103403.38 | 103405.05 | 103472.92 | 104099.45 | 1.67 | 67.87 | 626.53 | 696.07 |

Figure S3 shows that land transfer occurred across the whole province, especially in the southern region and the region near the coastline. The transferred grid in the southern is more intensive and concentrated, as this is where the conversion of cropland to built-up land mainly occurred. The coastline area always shows a much larger transferred land block, and this is where the transfer-out of grassland mainly occurred. The transfer-out of woodland is mainly distributed to the southwest and north of Jiangsu. Water area conversion is distributed across the whole study area, while the transfer-out of built-up land to other land use types occurred more in the north.


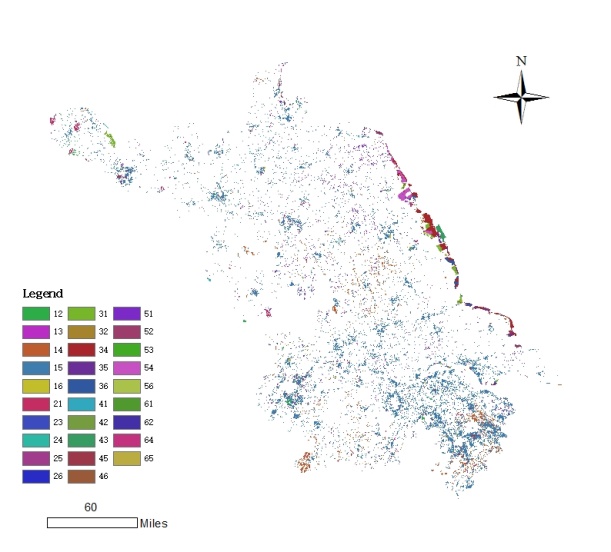


Figure S3 Spatial distribution of transferred land area between 1995 and 2010

Numbers 1-6 represent cropland, woodland, grassland, water area, built-up land and unused land. Code “12” represents the transfer of cropland to woodland, et cetera. Map created using ArcGIS [9.3], (http://www.esri.com/software/arcgis)

# SI-7. Carbon emissions from different energy sources

Carbon emissions from different energy sources all increased between 1995 and 2013. Coal consumption was always the main carbon source between 1995 and 2013; it accounted for 65.51% of total carbon emissions in 1995, and the percentages decreased gently, the lowest value of 42.06% appeared in 2012, and with a slight increase in 2013 to 44.49%. Electricity was the second-highest energy source of Jiangsu’s carbon emissions; before 2003 its percentages were a little lower than petroleum, while after 2003 it began to surpass petroleum and kept increasing, reaching about 30% in recent years. The percentages of petroleum did not change much, increasing before 2003 but decreasing gently after 2003, with the percentages varying between 13.91%-23.5% among different years. The amounts of carbon emissions from heat and natural gas increased the most, while compared with other sources, these percentages were still much lower, especially for natural gas, for which the highest percentage value was only 3.17% among different years.


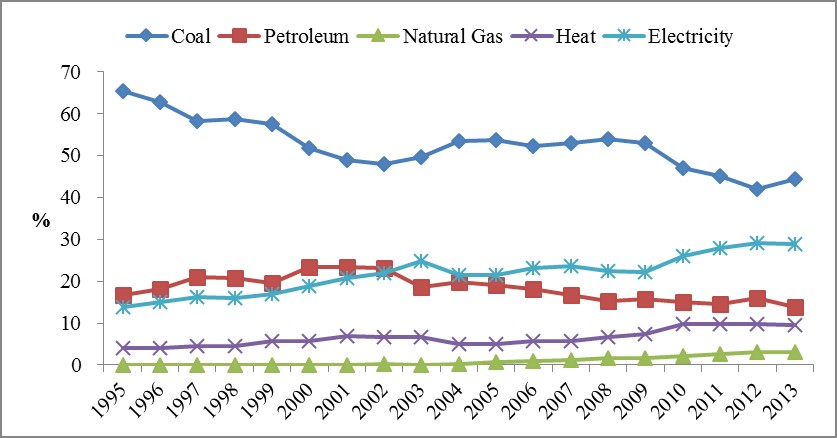


Figure S4 Percentages of carbon emissions from different energy sources

# References:

1. Chuai, X. W., Huang, X. J., Wang, W. J., Wu, C. Y. & Zhao, R. Q. Spatial simulation of land use based on terrestrial ecosystem carbon storage in coastal Jiangsu, China. *Sci. Rep-UK****.*** **4**, 5667 (2014).
2. Energy Statistics Division of the National Bureau of Statistics. China Energy Statistical Yearbook. China Statistics Press: Beijing, 1996- 2014. (in Chinese)
3. Intergovernmental Panel on Climate Change (IPCC).2006.National Greenhouse Gas Inventories Programme. In: Eggleston, H.S., et al. (Eds.), 2006 IPCC Guidelines for National Greenhouse Gas Inventories. Institute for Global Environmental Strategies, Hayama, Japan.
4. Liao, Q. L. *et al.* Increase in soil organic carbon stock over the last two decades in China's Jiangsu Province. *Global. Change. Biol.* **15**, 861-875 (2014).
5. Piao, S. L. *et al.* The carbon balance of terrestrial ecosystems in China. *Nature.* 458(7241), 1009-1013 (2009).
6. Xu, X. L., Cao, M. K. & Li, K. R. Temporal-spatial dynamics of carbon storage of forest vegetation in China. *Prog. Geogr.* **26**, 1-10 (2007).(in Chinese).
7. Zong, S. X. *et al.* A study of the biomass and energy of salt vegetation and sand vegetation alone the coast of Jiangsu Province. *J. Plant. Res. Environ*. **1**, 25-30 (1992).(in Chinese).
8. Zhao, R. Q. Carbon cycle of urban eco-economic system and its regulation through land use control: A case study of Nanjing city. Ph.D. Dissertation, Nanjing: Nanjing University, pp.54 (1992). (in Chinese).
